# HBx Enhances the Nrf2/ARE-dependent Antioxidant Response by Promoting Nuclear Export and Downregulation of Bach1

**DOI:** 10.24546/0100505589

**Published:** 2026-08-31

**Authors:** MUCHAMAD RIDOTU SOLICHIN, LIN DENG, HIFDZA FAZA FELISHA, YESHUA PUTRA KRISNUGRAHA, DEWA NYOMAN MURTI ADYAKSA, AULIANA HAYU KUSUMASTUTI, SAFIRA MADANIYAH, CHIEKO MATSUI, IKUO SHOJI

**Affiliations:** 1 Division of Infectious Disease Control, Center for Infectious Diseases, Kobe University Graduate School of Medicine, Kobe, Japan; 2 Faculty of Medicine, Public Health and Nursing, Universitas Gadjah Mada, Yogyakarta, Indonesia

**Keywords:** Hepatitis B virus, HBx, Bach1, Nrf2, NQO1, Antioxidant response

## Abstract

Hepatitis B virus (HBV) infection disrupts redox homeostasis in hepatocytes and is associated with the development of liver diseases. We previously reported that HBV activates the nuclear factor erythroid 2-related factor 2 (Nrf2)/antioxidant response element (ARE) signaling pathway, a major cellular defense mechanism against oxidative stress. However, it remains unclear whether Bach1, a transcriptional repressor that antagonizes Nrf2, is involved in the HBV-induced activation of Nrf2/ARE pathway. In this study, we aimed to elucidate how HBV affects Bach1 expression and subcellular localization and how these changes contribute to activation of the Nrf2/ARE signaling pathway. ARE reporter assays revealed that Bach1 knockdown significantly enhanced HBx-induced ARE activity, whereas Bach1 overexpression markedly attenuated it, indicating that Bach1 suppresses the HBx-mediated antioxidant response. HBV infection and HBx expression reduced endogenous Bach1 protein levels without affecting Bach1 mRNA levels, suggesting post-transcriptional downregulation. Immunofluorescence and subcellular fractionation suggested that HBx contributes to HBV-induced Bach1 nuclear export. Co-immunoprecipitation and immunofluorescence staining further demonstrated that HBx interacts with Bach1 in both the nucleus and cytoplasm. Notably, HBV infection upregulated the expression of NQO1, an Nrf2 target gene that was previously reported to contribute to HBx stabilization. In addition, Bach1 knockdown increased NQO1 and HBx protein levels. These findings suggest that Bach1 exerts an antiviral function by limiting Nrf2/ARE pathway activation and consequently restricting HBx stabilization. Collectively, our results suggest that HBx promotes nuclear export and downregulation of Bach1, thereby shifting the redox regulatory balance toward Nrf2 dominance, enhancing NQO1 expression, and stabilizing HBx protein.

## INTRODUCTION

Hepatitis B virus (HBV) infection remains a major global health burden and is a leading cause of chronic hepatitis, cirrhosis and hepatocellular carcinoma worldwide (1, 2). HBV belongs to the Orthohepadnavirus genus of the Hepadnaviridae family. HBV possesses a partially double-stranded circular DNA genome of approximately 3.2 kb. Following entry into hepatocytes, HBV particles are uncoated in the cytoplasm, and the relaxed circular DNA is transported to the nucleus, where it is converted into covalently closed circular DNA (cccDNA). This episomal cccDNA serves as the transcriptional template for viral RNAs of approximately 3.5 kb, 2.4 kb, 2.1 kb, and 0.7 kb. The 3.5-kb RNA encodes the HBc protein and HBV polymerase and also functions as pregenomic RNA (pgRNA), which is packaged together with polymerase into nucleocapsids and reverse-transcribed to generate the partially double-stranded DNA genome. The 2.4-kb and 2.1-kb RNAs encode the large, middle, and small HBs proteins, whereas the 0.7-kb RNA encodes HBx protein. Mature nucleocapsids are subsequently enveloped and secreted as infectious virions (3, 4).

Chronic HBV infection is associated with increased production of reactive oxygen species (ROS), resulting in oxidative DNA damage and lipid peroxidation in the liver (5, 6). Meanwhile, HBV replication induces the expression of antioxidant enzymes, including NAD(P)H quinone dehydrogenase 1 (NQO1), glutathione synthetase (GSS), and glutamate-cysteine ligase catalytic subunit (GCLC), suggesting that HBV simultaneously activates antioxidant defenses (7). Therefore, disruption of the balance between oxidative stress and antioxidant responses may contribute to HBV-associated liver diseases.

The transcription factor nuclear factor erythroid 2–related factor 2 (Nrf2), a member of the cap’n’collar basic leucine zipper (CNC-bZip) family, plays a central role in cellular antioxidant response. Under basal conditions, Nrf2 is targeted for ubiquitin-dependent proteasomal degradation by the Kelch-like ECH-associated protein 1 (Keap1)–Cullin3 complex. Upon oxidative stress conditions, Keap1 becomes inactivated, allowing Nrf2 to accumulate and translocate to the nucleus, where it binds antioxidant response elements (AREs) and induces the transcription of antioxidant genes (8). We previously reported that Keap1 activates the Nrf2/ARE signaling pathway through an interaction between HBx and Keap1. We also showed that activated Nrf2 suppresses the activities of HBV promoters and viral replication (9).

BTB and CNC homology 1 (Bach1) is another CNC-bZip transcription factor that represses antioxidant gene expression by competing with Nrf2 for binding to AREs under steady-state conditions (10). Under oxidative stress, stabilized Nrf2 translocates into the nucleus and functionally antagonizes Bach1-mediated repression, thereby allowing Nrf2-dependent transcriptional activation of antioxidant genes (11, 12). Despite the importance of the Nrf2/Bach1 regulatory axis in oxidative stress responses, it remains unclear whether HBV infection modulates Bach1 expression or subcellular localization.

In this study, we investigated the effects of HBV infection on Bach1 expression and localization and examined how these changes influence activation of the Nrf2/ARE pathway. Our findings suggest that HBV promotes Bach1 nuclear export and downregulation, thereby contributing to activation of the Nrf2/ARE signaling pathway and increase of HBx protein levels via NQO1 induction. These results identify Bach1 as a negative regulator of the HBV-induced antioxidant response and provide new insights into the interplay between HBV and host redox regulatory mechanisms.

## MATERIALS AND METHODS

### Cell culture

Human hepatoblastoma HepG2 cells were cultured in Eagle’s minimum essential medium (EMEM) containing L-glutamine (FUJIFILM Wako Pure Chemical Industries, Osaka, Japan) and supplemented with 10% heat-inactivated fetal bovine serum (FBS) (Biowest, Nuaillé, France), 100 U/mL penicillin, 100 μg/mL streptomycin (Gibco, Grand Island, NY, USA), and 0.1 mM nonessential amino acids (Gibco) at 37°C in a 5% CO_2_ incubator. HepG2-NTCP-Myc-His_6_ cells stably expressing sodium taurocholate cotransporting polypeptide (NTCP) (13) were maintained in Dulbecco’s modified Eagle’s medium (DMEM; high glucose) with L-glutamine (FUJIFILM Wako Pure Chemical Industries) supplemented with 10% FBS (Biowest), 10 mM HEPES (Gibco), 100 U/mL penicillin, 100 μg/mL streptomycin (Gibco), 0.1 mM nonessential amino acids (Gibco), and 1 ng/mL puromycin (Sigma-Aldrich, St. Louis, MO, USA). PXB primary human hepatocytes (PhoenixBio, Hiroshima, Japan) were cultured in dHCGM medium supplied by the manufacturer. Plasmid transfections were performed using FuGene 6 transfection reagent (Promega, Madison, WI, USA) following the manufacturer’s protocol.

### HBV preparation and infection

HBV genotype D stocks were prepared from the culture supernatant of Hep38.7-Tet cells as described previously (13). Briefly, the culture supernatants were clarified by centrifugation, and HBV particles were precipitated overnight at 4°C with 12.5% (wt/vol) polyethylene glycol 6000 (PEG-6000) (Hampton Research, Aliso Viejo, CA, USA) and 0.75 M NaCl. The pellet was collected by centrifugation at 9,100 × g for 60 min at 4°C, resuspended, and further purified through a 20% sucrose cushion by ultracentrifugation at 110,000 × g for 16 h at 4°C using an SW41 Ti rotor (Beckman Coulter, Brea, CA, USA). Purified HBV was resuspended in Opti-MEM (Thermo Fisher Scientific, Waltham, MA, USA) and stored at −80°C. HBV genome equivalents (GEq) were quantified by quantitative PCR (qPCR), using pUC19-HBV-D-IND60 as a standard. HBV infection was performed as described previously (14). HepG2-NTCP-Myc-His_6_ cells or PXB cells were inoculated with HBV at 1,000 GEq/cell in the presence of 4% PEG-8000 at 37°C for 24 h. The cells were then washed twice with medium and cultured in fresh medium.

### Expression plasmids

The Bach1 expression plasmid was generated using Bach1 cDNA obtained from Huh-7.5 cells. The cDNA fragment was inserted into the NotI site of pCAG-FLAG using the In-Fusion HD Cloning Kit (Clontech, Mountain View, CA, USA), resulting in the construct pCAG-FLAG-Bach1. The sequence of the pCAG-FLAG-Bach1 was confirmed by DNA sequencing (Eurofins Genomics, Tokyo, Japan). The HBV plasmid pUC19-HBV-C-AT_JPN carries a 1.3-mer overlength HBV genome (15). The expression plasmids pEF1A-HBx-Myc-His_6_, pEF1A-HBc-Myc-His_6_, pEF1A-LHBs-Myc-His_6_, pEF1A-HBV Pol-Myc-His_6_ (16), and pEGFP-C3-HBx (9) were used in this study.

### Antibodies and reagents

The mouse monoclonal antibodies (MAbs) used in this study were anti-Bach1 (F-9; sc-271211; Santa Cruz Biotechnology, Santa Cruz, CA, USA), anti-FLAG (M2) (F3165; Sigma-Aldrich, Darmstadt, Germany), anti-c-Myc MAb (9E10; sc-40; Santa Cruz Biotechnology), anti-HBc MAb (clone 7B2, hybridoma culture supernatant) (17), anti-HBc MAb (MA1-7607; Invitrogen, Carlsbad, CA, USA), anti-Histone H3 (1G1) MAb (sc-517576; Santa Cruz Biotechnology), anti-NQO1 (A180; sc-32793; Santa Cruz Biotechnology), and anti-glyceraldehyde-3-phosphate dehydrogenase (GAPDH) MAb (014-25524; FUJIFILM Wako Pure Chemical Industries). The rabbit polyclonal antibodies (PAbs) used in this study were anti-Bach1 rabbit PAb (PA5-103060; Thermo Fisher Scientific), anti-HBx PAb (ab39716; Abcam, Cambridge, UK), and anti-IκBα PAb (9242; Cell Signaling Technology, Danvers, MA, USA). Horseradish peroxidase (HRP)-conjugated anti-mouse IgG (7076; Cell Signaling Technology) and HRP-conjugated anti-rabbit IgG (7074; Cell Signaling Technology) were used as secondary antibodies. The NQO1 inhibitor, Dicoumarol (CAS 66-76-2, Merck, Darmstadt, Germany), was dissolved in 0.1 N NaOH.

### Luciferase reporter assay

The plasmid pGL4.37[luc2P/ARE/Hygro] (Promega) contains four copies of an antioxidant response element (ARE) that drives transcription of the firefly luciferase reporter gene luc2P. The firefly luciferase reporter plasmids carrying the entire HBV Ce core promoter (nt 900 to 1851; a nonsense mutation [ATG to TAG at nt 1374 to 1376] was introduced at the start codon of the HBx gene), Enh1/X promoter (nt 950 to 1373), preS1 promoter (nt 2707 to 2847), or preS2/S promoter (nt 2937 to 3204) were described previously (16). The plasmid pRL-CMV-*Renilla* (Promega), which expresses *Renilla* luciferase, was used as an internal control. HepG2 cells cultured in 24-well plates were transiently transfected with the indicated reporter plasmids. At 48 h after transfection, the cells were harvested and luciferase activities were measured using a dual-luciferase reporter assay system (Promega) with a GloMax 96 microplate luminometer (Promega). Firefly luciferase activity was normalized to *Renilla* luciferase activity for each sample.

### siRNA transfection

HepG2, HepG2-NTCP-Myc-His_6_, or PXB cells were transfected with Bach1 siRNA (SMARTpool; Dharmacon, Lafayette, CO, USA) at a final concentration of 40 nM using Lipofectamine RNAiMAX (Thermo Fisher Scientific) according to the manufacturer’s instructions. AllStars Negative Control siRNA (Qiagen) was used as a control.

### Immunoprecipitation

Cultured cells were lysed for 30 min on ice in lysis buffer containing 150 mM NaCl, 50 mM Tris-HCl (pH 7.5), 1 mM EDTA (pH 8.0), 0.1% sodium dodecyl sulfate (SDS), 1% sodium deoxycholic acid, 1% Triton X-100, and protease inhibitor cocktail (Roche). The lysates were cleared by centrifugation at 20,400 × g for 15 min at 4°C. The supernatants were subjected to immunoprecipitation with anti-FLAG M2 affinity gel (Sigma-Aldrich) or protein G-Sepharose 4 Fast Flow (GE17-5280-04; GE Healthcare, Buckinghamshire, UK) pre-coupled with the indicated antibody overnight at 4°C with rotation. The beads were washed five times with lysis buffer, and the bound proteins were eluted by boiling in SDS sample buffer.

### Immunoblot analysis

Immunoblot analysis was performed as described previously (16). Proteins were separated by SDS-polyacrylamide gel electrophoresis (SDS-PAGE) and transferred to a polyvinylidene difluoride membrane (Millipore, Billerica, MA, USA). The membranes were incubated with primary antibodies, followed by horseradish peroxidase (HRP)-conjugated secondary antibodies. Protein bands were visualized using enhanced chemiluminescence western blotting detection reagents (ECL; GE Healthcare). Band intensities were quantified by densitometric analysis using ImageJ software (version 1.56d). The densitometric quantification of target proteins was normalized to the corresponding loading controls.

### RNA extraction and quantitative reverse transcription-PCR (RT-qPCR)

Total RNA was extracted using the ReliaPrep RNA Cell Miniprep System (Promega) following the manufacturer’s instructions, and cDNA was synthesized using GoScript Reverse Transcription (Promega) with random hexamer primers. qPCR was performed using TB Green Premix Ex Taq II (TaKaRa Bio, Kyoto, Japan) with SYBR green chemistry on a StepOnePlus real-time PCR System (Applied Biosystems, Foster City, CA, USA). The primer sequences were as follows: HBV total RNA (amplifying all HBV transcripts except the 0.8-kb transcript encoding HBx) (18), forward, 5′-GCTTTCACTTTCTCGCCAAC-3′; reverse, 5′-GAGTTCCGCAGTATGGATCG-3′; HBV pgRNA (19), forward, 5′-ACTGTTCAAGCCTCCAAGCTGT-3′; reverse, 5′-GAAGGCAAAAACGAGAGTAACTCCAC-3′; Bach1, forward, 5′-CGCCTCAGCTCTGGTTGATG-3; reverse, 5′-CATCAGCCTGGCCTACGATT-3′. NQO1, forward 5′-GGGCAAGTCCATCCCAACTG-3′ and reverse 5′-GCAAGTCAGGGAAGCCTGGA-3′; Human GAPDH mRNA levels were measured as an internal control, using the primers 5′-GCCATCAATGACCCCTTCATT-3′ and 5′-TCTCGCTCCTGGAAGATGG-3′.

### Indirect immunofluorescence

Indirect immunofluorescence staining was performed as described previously (16). Briefly, HepG2 cells grown on glass coverslips placed in a 24-well plate were fixed with 4% paraformaldehyde for 15 min at room temperature, permeabilized with 0.1% Triton X-100 in PBS for 15 min at room temperature, and blocked with 5% bovine serum albumin (BSA) in PBS for 30 min. After being washed twice with PBS, the cells were incubated with primary antibodies, followed by secondary antibodies. The primary antibodies used were anti-Bach1 rabbit PAb (PA5-103060; Thermo Fisher Scientific), anti-c-Myc mouse MAb (9E10; sc-40; Santa Cruz Biotechnology), and anti-HBc mouse MAb (MA1-7607, Invitrogen). The secondary antibodies used were Alexa Fluor 594-conjugated goat anti-mouse IgG (A11005; Molecular Probes, Eugene, OR, USA), Alexa Fluor 488-conjugated goat anti-rabbit IgG (A11008; Molecular Probes), and Alexa Fluor 647-conjugated goat anti-rabbit IgG (ab150079; Abcam). The stained cells were counterstained with Hoechst 33342 (Molecular Probes) and observed using a confocal laser scanning microscope (LSM700; Carl Zeiss, Oberkochen, Germany).

### Proximity ligation assay (PLA)

In situ PLA was performed using the Duolink In Situ PLA kit (Sigma-Aldrich) as described previously (20). To evaluate the interaction between Bach1 and HBx, HepG2 cells cultured on glass coverslips in 24-well plates were co-transfected with pEF1A-HBx-Myc-His_6_ and pCAG-FLAG-Bach1. At 48 h after transfection, the cells were fixed with 4% paraformaldehyde for 15 min and permeabilized with PBS containing 0.2% Triton X-100 for 15 min at room temperature. The coverslips were incubated with anti-c-Myc mouse MAb and anti-Bach1 rabbit PAb. The samples were washed three times with the wash buffer supplied in the kit. PLA probes (anti-mouse MINUS and anti-rabbit PLUS) were diluted in the supplied antibody diluent, and the samples were incubated for 1 h at 37°C in a humidity chamber. The samples were washed and processed according to the manufacturer’s instructions for probe ligation, signal amplification, and mounting. Fluorescent PLA signals were examined using a confocal laser scanning microscope (LSM 700; Carl Zeiss).

### Extracellular HBsAg quantification (ELISA)

Culture supernatants were clarified by centrifugation at 1,000 × g for 5 min at 4°C. Extracellular HBsAg levels were quantified using an HBs ELISA kit (DIAsource, Louvain-la-Neuve, Belgium) according to the manufacturer’s instructions.

### Subcellular fractionation

Cytoplasmic and nuclear fractions were prepared from HepG2 cells using the ProteoExtract Subcellular Proteome Extraction Kit (539790; Sigma-Aldrich) according to the manufacturer’s instructions. The resulting lysates were subsequently analyzed by immunoblotting. IκBα and histone H3 were used as cytoplasmic and nuclear fraction markers, respectively.

### Cell viability assay

Cell viability was evaluated by measuring intracellular ATP levels using the CellTiter-Glo v2.0 assay kit (Promega) according to the manufacturer’s instructions. Luminescence signals were measured using a GloMax Navigator microplate luminometer GM2000 (Promega).

### Database

Transcriptomic data from patients with chronic HBV infection were obtained from the Gene Expression Omnibus (GEO) public database (accession number GSE83148). This dataset was generated by microarray expression profiling using the Affymetrix Human Genome U133 Plus 2.0 Array platform and included liver tissue samples from patients with chronic HBV infection (n = 122) and healthy controls (n = 6). NQO1 expression levels were extracted for analysis. Data normality was assessed using the Kolmogorov–Smirnov and Shapiro–Wilk tests. As the data were not normally distributed, group comparisons between groups were performed using the Mann–Whitney U test. A P-value < 0.05 was considered statistically significant.

### Statistical analysis

All quantitative data are presented as the means ± standard errors of the mean (SEM) from at least three independent experiments. Two-group comparisons were performed using a two-tailed Student’s t-test. A P value < 0.05 was considered statistically significant.

## RESULTS

### Bach1 suppresses HBx-mediated activation of the antioxidant response

To evaluate the functional role of Bach1 in the HBx-mediated antioxidant response, ARE-driven luciferase reporter assays were performed in HepG2 cells. Cells were co-transfected with pEF1A-HBx-Myc-His_6_ and pGL4.37[luc2P/ARE/Hygro], together with either control or Bach1 siRNAs. The luciferase reporter assay showed that HBx expression markedly increased ARE-luciferase activity, and siRNA-mediated knockdown of Bach1 further enhanced this HBx-induced activation (Fig. 1A, left panel). The efficiency of Bach1 knockdown was confirmed by immunoblotting (Fig. 1A, right panel). Conversely, exogenous expression of FLAG-Bach1 significantly reduced HBx-mediated ARE-luciferase activity (Fig. 1B, left panel). Immunoblot analysis confirmed FLAG-Bach1 expression (Fig. 1B, right panel). These results suggest that Bach1 negatively regulates the HBx-mediated antioxidant response.

### HBV infection reduces endogenous Bach1 protein levels

To investigate whether HBV infection affects Bach1 expression, we examined Bach1 mRNA and protein levels in HBV-infected cells by RT-qPCR and immunoblot analysis, respectively. In HBV-infected HepG2-NTCP-Myc-His_6_ cells, Bach1 mRNA levels were comparable to those in mock-infected cells (Fig. 2A). However, endogenous Bach1 protein levels were reduced following HBV infection (Fig. 2B, upper panel, lane 2). Importantly, a similar reduction in endogenous Bach1 protein was observed in HBV-infected PXB cells, primary human hepatocytes derived from humanized chimeric mouse livers (Fig. 2C, upper panel, lane 2). Collectively, these findings demonstrate that HBV infection reduces Bach1 protein levels without affecting Bach1 mRNA expression.

To determine which HBV protein is responsible for the HBV-induced reduction in endogenous Bach1 protein levels, we next examined the effect of HBx on endogenous Bach1 expression. HepG2 cells were transfected with either pEF1A-HBx-Myc-His_6_ or a control vector. Immunoblot analysis showed that endogenous Bach1 protein levels were reduced in HBx-expressing cells (Fig. 2D, upper panel, lane 2), whereas HBc, LHBs, and polymerase had no appreciable effects (Fig. 2E, upper panel). These results suggest that HBx contributes to the post-transcriptional downregulation of Bach1 during HBV infection.

### HBV infection and HBx expression promotes the nuclear export of Bach1

To determine whether HBV infection affects the subcellular localization of Bach1, we conducted indirect immunofluorescence staining using HBV-infected HepG2-NTCP-Myc-His_6_ cells. The cells were fixed and analyzed at 12 dpi. Representative images showed Bach1 signals in HBV-infected (HBc-positive) cells were predominantly enriched in the cytoplasm compared with the nuclear localization observed in mock-infected cells (Fig. 3A, bottom row, second panel from the left). These findings suggest that HBV infection promotes the translocation of Bach1 from the nucleus to the cytoplasm.

Next, to assess the effect of HBx on Bach1 subcellular localization, we performed subcellular fractionation. Immunoblot analysis revealed increased cytoplasmic accumulation of Bach1 in HBx-expressing cells (Fig. 3B, top panel, compare lanes 1 and 2; Fig. 3C), accompanied by a decrease in nuclear Bach1 (Fig. 3B, top panel, compare lanes 3 and 4; Fig. 3C). These results suggest that HBx promotes Bach1 nuclear export and may contribute to HBV-induced Bach1 redistribution.

### HBx interacts with Bach1 in both the nucleus and the cytoplasm

To investigate the mechanism underlying HBx-mediated nuclear export of Bach1, we first examined whether HBx physically interacts with Bach1 by co-immunoprecipitation analysis. Co-transfection of pCAG-FLAG-Bach1 and pEF1A-HBx-Myc-His_6_ in HepG2 cells, followed by immunoprecipitation with an anti-FLAG antibody and immunoblotting with an anti-c-Myc antibody, demonstrated HBx-Myc-His_6_ was co-immunoprecipitated with FLAG-Bach1 (Fig. 4A, top panel, lane 4).

To investigate the interaction between endogenous Bach1 and HBx, HepG2 cells were transfected with pEF1A-HBx-Myc-His_6_. Immunoprecipitation analysis using an anti-c-Myc antibody showed that endogenous Bach1 was co-immunoprecipitated with HBx-Myc-His_6_ (Fig. 4B, top panel, lane 2), whereas a non-specific IgG control showed no signal (Fig. 4B, top panel, lane 1). These results suggest that HBx interacts with endogenous Bach1.

To determine the subcellular localization of endogenous Bach1 and HBx, we performed indirect immunofluorescence staining. HepG2 cells were transfected with pEF1A-HBx-Myc-His_6_ or pEF1A-Myc-His_6_. In control cells, endogenous Bach1 was localized in both the nucleus and the cytoplasm (Fig. 4C, top panel, Bach1). In HBx-Myc-His_6_-expressing cells, nuclear localization of Bach1 was reduced (Fig. 4C, bottom panel, Bach1). This observation is consistent with the results shown in Fig. 3B, suggesting that HBx promotes Bach1 nuclear export. Moreover, the merged yellow signals indicated colocalization of HBx-Myc-His_6_ and Bach1 in both the nucleus and the cytoplasm (Fig. 4C, bottom panel, merge).

To further examine whether Bach1 colocalizes with HBx, we performed PLA to detect protein-protein interactions as fluorescent signals in cells. HepG2 cells were co-transfected with pCAG-FLAG-Bach1 and pEF1A-HBx-Myc-His_6_. PLA revealed strong fluorescent signals in both the nucleus and the cytoplasm when FLAG-Bach1 and HBx-Myc-His_6_ were co-expressed (Fig. 4D, bottom panel, merge). These results suggest that HBx interacts with Bach1 in both the nucleus and the cytoplasm.

### Knockdown of Bach1 enhances HBV replication and increases HBx protein levels

To assess the role of Bach1 in HBV life cycle, HBV-infected PXB cells were transfected with Bach1 siRNA. Knockdown of Bach1 significantly enhanced intracellular HBV RNA and pgRNA levels (Fig. 5A), as well as HBc protein levels (Fig. 5B, second panel, lane 2). Similarly, in HepG2 cells transfected with plasmid pUC19-HBV-CAT_JPN, Bach1 siRNA-mediated knockdown of Bach1 significantly increased intracellular HBV RNA levels (Fig. 5C), HBx protein levels (Fig. 5D, second panel, lane 2), and HBc protein levels (Fig. 5D, third panel, lane 2). In addition, Bach1 knockdown increased extracellular HBsAg levels (Fig. 5E). These findings suggest that Bach1 plays an antiviral role in HBV replication.

To further examine the effect of Bach1 on HBx protein expression, HepG2 cells were transfected with pEF1A-HBx-Myc-His_6_ in the presence or absence of Bach1siRNA. Immunoblot analysis showed that Bach1 knockdown increased HBx protein levels (Fig. 5F, second panel, lane 2). Collectively, these results suggest that Bach1 functions as a host restriction factor that suppresses HBV replication, at least in part by limiting HBx protein levels.

### Bach1 does not directly affect HBV promoter activities

To determine whether Bach1-mediated inhibition of HBV replication is due to direct regulation of HBV promoters, we next examined the effects of Bach1 knockdown or overexpression on the activities of four different HBV promoters: core, preS1, preS2/S, and enhancer 1 [Enh1]/X, derived from HBV genotype Ce. HepG2 cells were co-transfected with either Bach1 siRNA or pCAG-FLAG-Bach1, together with a firefly luciferase reporter driven by the core, preS1, preS2/S, and Enh1/X promoters. Promoter reporter assays demonstrated that knockdown of Bach1 did not significantly affect the activities of the core, preS1, preS2/S, or Enh1/X promoters (Fig. 6A). Similarly, overexpression of FLAG-Bach1 also had no significant effects on these HBV promoter activities (Fig. 6B). These results suggest that Bach1 does not regulate HBV transcription through modulation of HBV promoter activities.

### HBV-induced Bach1 downregulation is associated with NQO1 upregulation and increased HBx protein levels

To determine whether HBV-induced Bach1 downregulation and nuclear export affect downstream gene expression, we focused on a representative Bach1 target gene, NAD(P)H quinone oxidoreductase 1 (NQO1). We first analyzed transcriptomic data from liver tissue samples of patients with chronic HBV infection (GSE83148). Hepatic NQO1 expression levels were significantly upregulated in HBV-infected individuals relative to healthy controls (Fig. 7A). To assess whether Bach1 is involved in HBV-induced upregulation of NQO1, we knocked down Bach1 using siRNA in HepG2 cells transfected with pUC19-HBV-C-AT_JPN and in HBV-infected PXB cells. Following Bach1 depletion, NQO1 mRNA levels were significantly increased in both HBV-replicating cells (Fig. 7B) and HBV-infected PXB cells (Fig. 7C), suggesting that HBV-induced upregulation of NQO1 is associated with Bach1 downregulation and nuclear export.

To further investigate the potential relationship among Bach1, NQO1, and HBx, we examined the effect of Bach1 depletion on endogenous NQO1 expression in HBx-expressing cells. HepG2 cells were transfected with pEF1A-HBx-Myc-His_6_, with or without Bach1 siRNA. Bach1 knockdown increased both NQO1 protein (Fig. 7D, second panel, lane 2) and HBx protein levels (Fig. 7D, third panel, lane 2), raising the possibility that increased NQO1 expression contributes to HBx stabilization. To further determine whether the Bach1 depletion-induced increase in HBx protein levels is associated with increased NQO1 activity, we treated cells with the NQO1 inhibitor dicoumarol. Immunoblot analysis showed that Bach1 depletion-induced HBx enhancement was reduced by treatment with 25 μM dicoumarol (Fig. 7E, second panel, compare lanes 2 and 3), without affecting cell viability at this concentration (Fig. 7F). Collectively, these findings suggest that HBV-induced Bach1 downregulation is associated with increased NQO1 expression and HBx protein levels.

## DISCUSSION

HBV infection induces oxidative stress and activates antioxidant responses, thereby altering redox homeostasis in infected hepatocytes to create an intracellular environment favorable for viral persistence (21–23). In this study, we identified Bach1 as a regulator of the HBV–Nrf2 axis and demonstrated that it functions as a host antiviral factor that limits HBx accumulation. We found that Bach1 inhibited HBx-mediated antioxidant response (Fig. 1), whereas Bach1 knockdown increased HBx protein levels and enhanced HBV replication (Fig. 5). Conversely, HBV infection reduced Bach1 protein levels and promoted its nuclear export (Figs. 2 and 3A). We further demonstrated that HBx interacted with Bach1 (Fig. 4) and contributed to Bach1 nuclear export (Fig. 3B), suggesting that HBx relieves Bach1-mediated repression of antioxidant genes during HBV infection. Based on these findings, we propose a model in which HBx promotes Bach1 nuclear export and downregulation, thereby enhancing Nrf2-dependent expression of antioxidant genes, such as NQO1. Given that NQO1 stabilizes HBx protein, activation of the Nrf2–NQO1 pathway may establish a positive feedback loop that further promotes HBx accumulation and contributes to HBV replication (Fig. 8).

Bach1 stabilization is regulated by multiple cellular signals. Heme is a well-characterized negative regulator of Bach1. Upon binding to Bach1, heme promotes its nuclear export through a chromosome region maintenance 1 (CRM1)-dependent mechanism and induces ubiquitin-dependent degradation of Bach1 (24–26). Interestingly, HBx has been reported to interact with CRM1 and alter its subcellular distribution (27), raising the possibility that HBx may promote Bach1 nuclear export through a CRM1-dependent pathway. In addition, Bach1 nuclear export has been reported to be promoted by phosphorylation at tyrosine 486 (28). Therefore, it is possible that HBV-induced signaling pathways contribute to the regulation of Bach1 localization. Future studies will be required to determine whether CRM1 and Bach1 phosphorylation are involved in HBx-mediated Bach1 nuclear export.

Bach1 forms heterodimers with small Maf proteins and represses transcription of Nrf2 target genes through ARE binding (12, 24, 29, 30). Because Nrf2 and MafF have been shown to suppress HBV core promoter activity (9, 19), we initially hypothesized that Bach1 may also regulate HBV transcription. However, neither knockdown nor overexpression of Bach1 affected HBV promoter activity (9, 19), we initially hypothesized that Bach1 may also regulate HBV transcription. However, neither knockdown nor overexpression of Bach1 affected HBV promoter activity

Bach1 is a negative regulator of Nrf2-mediated NQO1 expression and directly competes with Nrf2 for binding to AREs, thereby repressing transcription of Nrf2 target genes (12). Transcriptomic analysis of HBV-infected liver tissues demonstrated increased NQO1 expression during HBV infection (Fig. 7A). Given that NQO1 has been reported to stabilize HBx by preventing its proteasomal degradation (31), HBV-induced Bach1 downregulation may contribute to increased HBx protein levels through NQO1 upregulation. However, NQO1 is only one of many Nrf2 target genes. Indeed, we previously reported that another Nrf2 target gene, Prdx1, suppresses HBV replication by promoting HBV RNA degradation (16). These observations highlight the complexity of Nrf2 signaling in HBV infection and suggest that the net effect of Nrf2/ARE activation depends on the balance among its downstream target genes. Further studies will be required to comprehensively evaluate how the Nrf2/ARE pathway influences HBV replication and persistence.

Several limitations of this study should be acknowledged. First, although our results indicate that HBx is associated with Bach1 nuclear export and downregulation, the molecular basis of these effects remains to be determined. In particular, it remains unclear whether specific nuclear export machinery, altered protein degradation pathways, or additional host cofactors are involved. Given that HBx has been reported to promote the proteasomal degradation of several host factors, including the structural maintenance of chromosomes (Smc) complex Smc5/6 (32) and zinc finger E-box binding homeobox 2 (ZEB2) (33), it will be important to determine whether HBx-dependent Bach1 degradation is also mediated by ubiquitination and subsequent proteasomal degradation. Second, some of the effects of Bach1 knockdown on HBV-related readouts were modest in magnitude, suggesting that Bach1 is likely one of multiple host factors contributing to the regulation of HBV-associated redox responses. Third, because the Nrf2 pathway regulates a broad range of downstream genes with potentially distinct effects on viral replication and cell survival, the overall impact of Nrf2/Bach1 modulation during HBV infection is likely to depend on cellular context and the balance of target genes induced.

In summary, our findings demonstrate that Bach1 acts as a negative regulator of the HBV-induced antioxidant response and that HBx is associated with Bach1 redistribution and downregulation during infection. HBx promotes Bach1 nuclear export and downregulation, thereby shifting the antioxidant balance toward Nrf2 dominance. This shift enhances NQO1 expression, thereby contributing to HBx stabilization. These findings provide insight into how HBV exploits host redox pathways and suggest that restoring redox homeostasis may represent a potential strategy for limiting HBV replication.

## Figures and Tables

**Fig.1:**
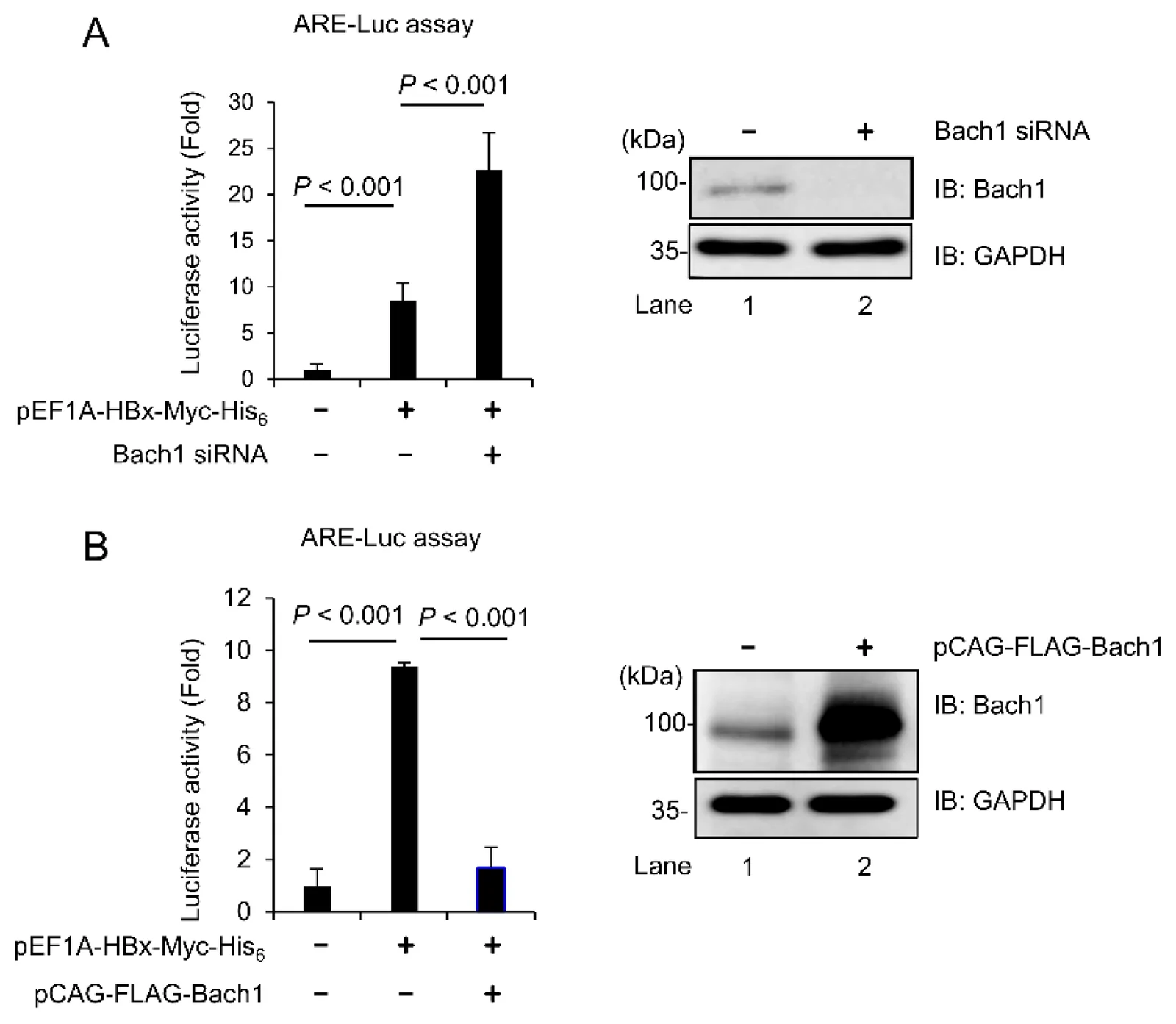
Bach1 suppresses the HBx-mediated antioxidant response (A) HepG2 cells were transfected with 40 nM Bach1 siRNA or control siRNA. At 24 h after siRNA transfection, the cells were co-transfected with pGL4.37[luc2P/ARE/Hygro] and pEF1A-HBx-Myc-His_6._ (B) HepG2 cells were co-transfected with pGL4.37[luc2P/ARE/Hygro] and pEF1A-HBx-Myc-His_6_, together with pCAG-FLAG-Bach1 or control plasmid. (A and B) At 48 h after plasmid transfections, the cells were harvested. ARE-luciferase activity was measured and normalized to *Renilla* activity. Bach1 expression in cell lysates were analyzed by immunoblotting with anti-Bach1 MAb. The level of GAPDH served as a loading control (A and B, right panels). Data represent the means ± SEM of data from three independent experiments. The value for the control cells was arbitrarily set to 1.0. compared with the controls. A P-value < 0.05 was considered statistically significant.

**Fig. 2:**
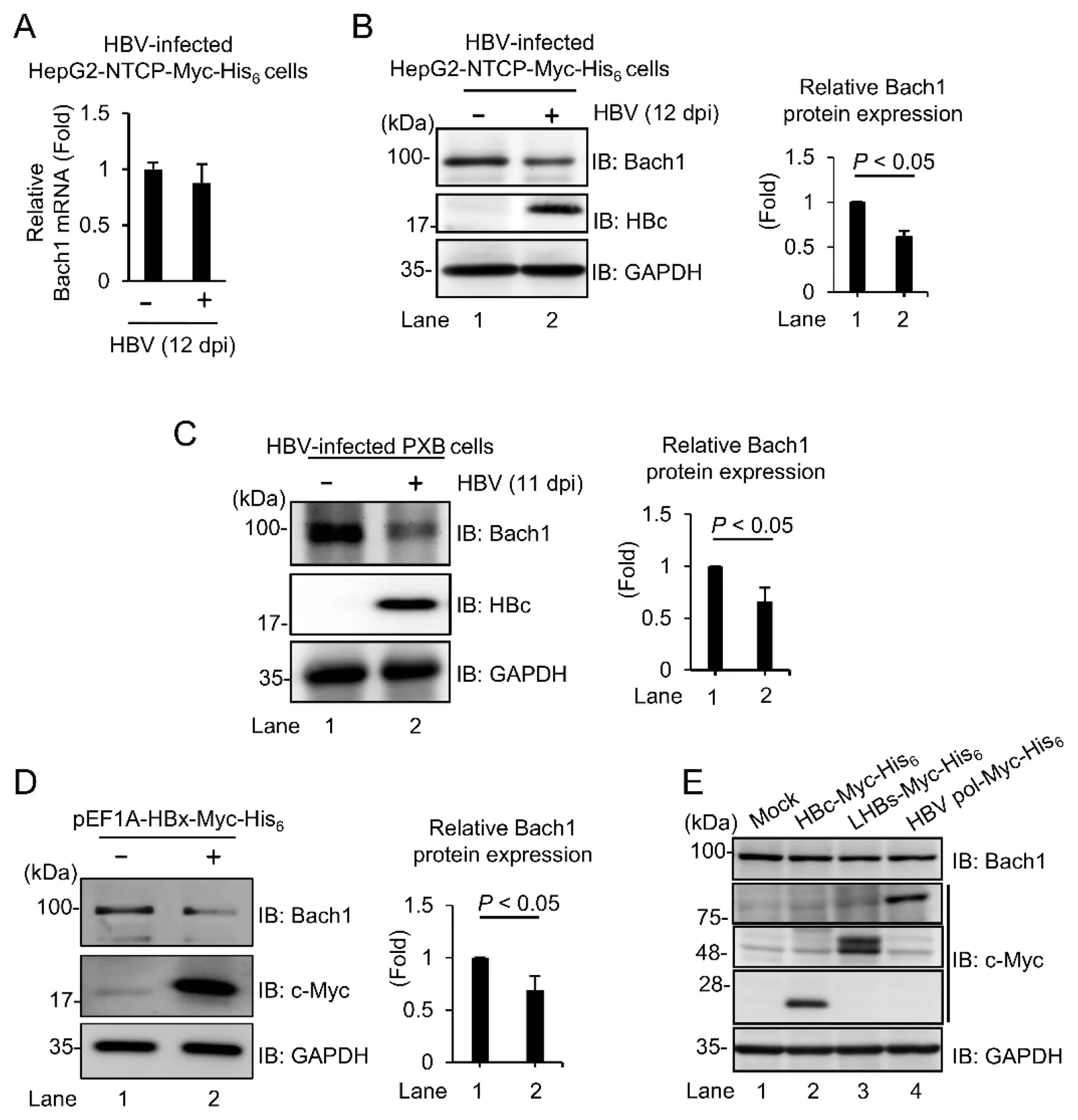
HBV infection and HBx expression reduce endogenous Bach1 protein levels (A and B) HepG2-NTCP-Myc-His_6_ cells were infected with HBV at 1,000 GEq/cell and harvested at 12 days post-infection (dpi). (A) Total RNA was extracted, and Bach1 mRNA was quantified by RT-qPCR. Data represent the means ± SEM from three biological replicates. Values for control cells were arbitrarily set to 1.0. A P-value < 0.05 was considered statistically significant. (B) Cell lysates were subjected to immunoblotting using anti-Bach1 MAb (first panel), anti-HBc MAb (second panel), and anti-GAPDH MAb (third panel), with GAPDH serving as the loading control. The relative levels of Bach1 were quantified by densitometric analysis from three independent experiments. (C) PXB cells were infected with HBV at 1,000 GEq/cell and harvested at 11 dpi. Cell lysates were subjected to immunoblotting as described in (B). (D and E) HepG2 cells were transfected with pEF1A-HBx-Myc-His_6_ (D), or with Myc-His_6_-tagged HBc, LHBs, or HBV polymerase (Pol) expression plasmids (E). At 48 h after plasmid transfection, the cells were harvested. The expression levels of endogenous Bach1 protein in cell lysates were analyzed by immunoblotting with anti-Bach1 MAb. The level of GAPDH served as a loading control. The relative levels of Bach1 were quantified by densitometric analysis from three independent experiments. A P-value < 0.05 was considered statistically significant.

**Fig. 3:**
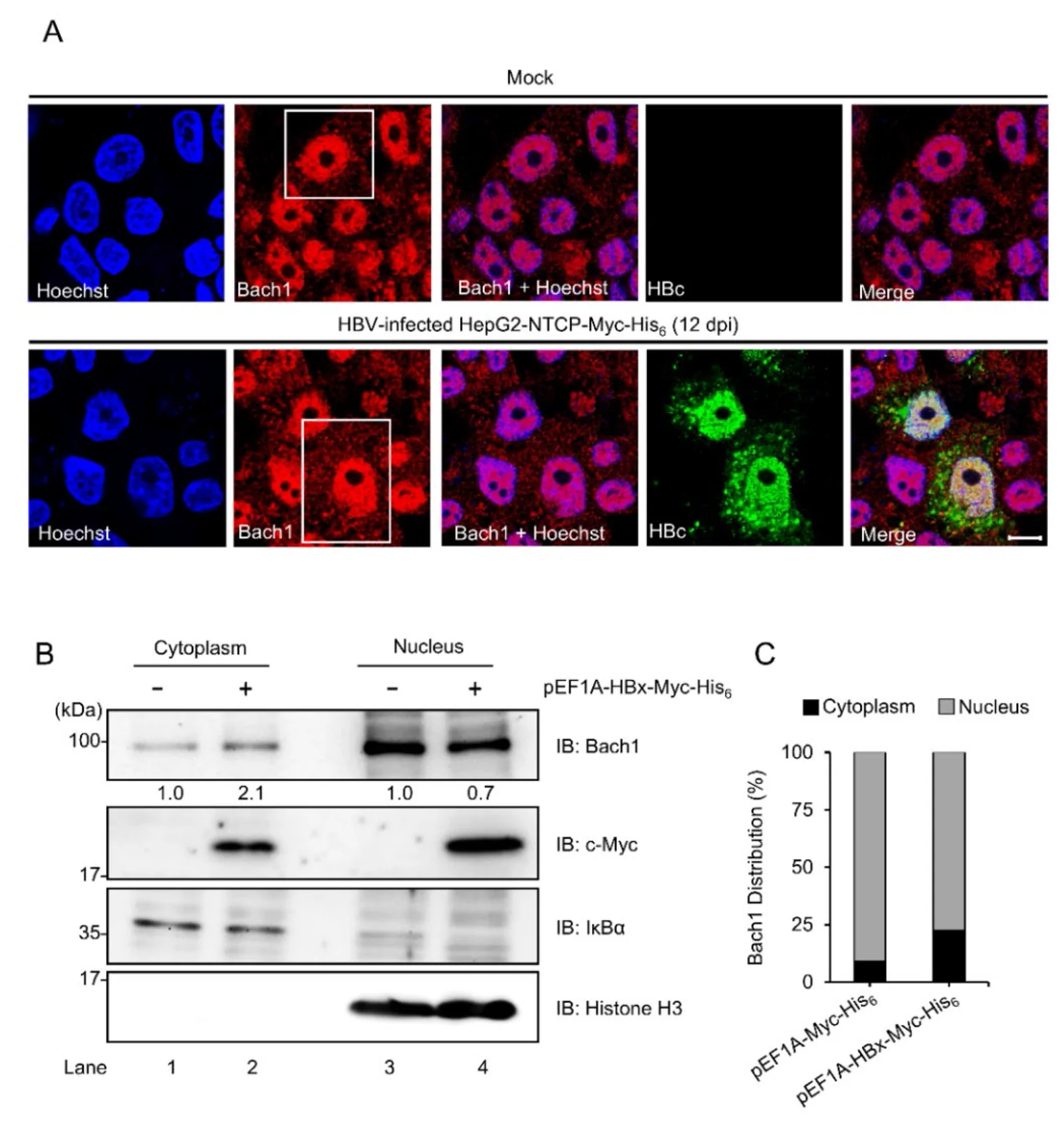
HBV infection promotes Bach1 nuclear export (A) HepG2-NTCP-Myc-His_6_ cells were infected with HBV at 1,000 GEq/cell. At 12 dpi, cells were fixed and subjected to indirect immunofluorescence staining using an anti-HBc mouse MAb followed by Alexa Fluor 488-conjugated goat anti-mouse IgG (green), and an anti-Bach1 rabbit PAb followed by Alexa Fluor 594-conjugated goat anti-rabbit IgG (red). Nuclei were counterstained with Hoechst 33342 (blue). Scale bar, 20 μm. (B and C) HepG2 cells were transfected with pEF1A-Myc-His_6_ or pEF1A-HBx-Myc-His_6_. At 48 h after transfection, cells were harvested and subjected to subcellular fractionation. (B) Cytoplasmic and nuclear fractions were analyzed by immunoblotting with the indicated antibodies. The relative levels of cytoplasmic and nuclear Bach1 were quantified by densitometry and are indicated below each lane. IκBα and histone H3 served as cytoplasmic and nuclear markers, respectively. (C) Stacked bar graph showing the relative distribution of Bach1 between the cytoplasm and nucleus. Data represent the means ± SEM of data from three independent experiments.

**Fig. 4:**
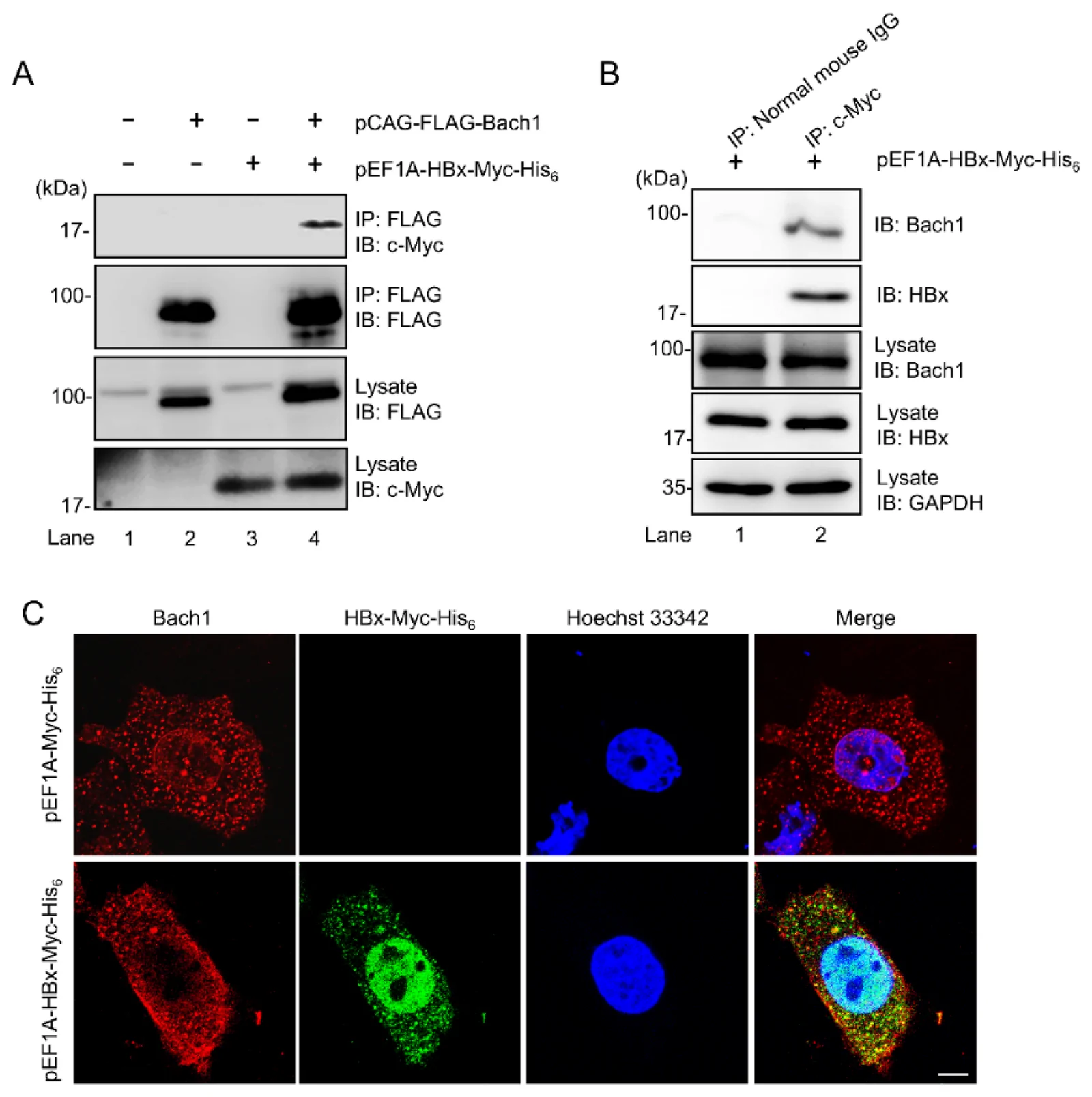

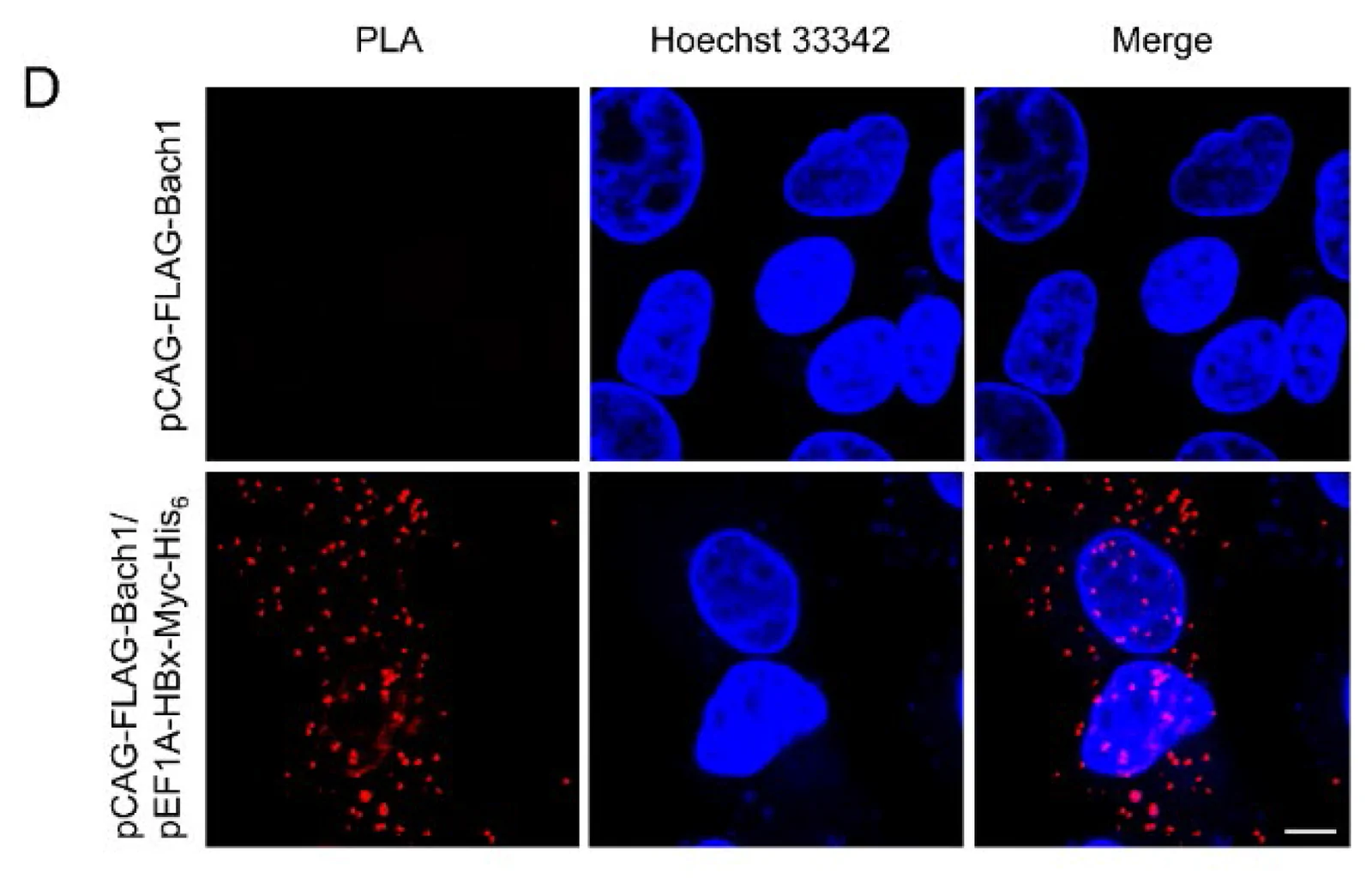
HBx physically interacts with Bach1 in both the nucleus and cytoplasm (A) HepG2 cells were co-transfected with pCAG-FLAG-Bach1 and pEF1A-HBx-Myc-His_6_. At 48 h after transfection, cells were harvested. Cell lysates were immunoprecipitated with anti-FLAG beads, followed by immunoblotting with anti-c-Myc MAb (top panel) or anti-FLAG MAb (second panel). Input samples were immunoblotted with anti-FLAG MAb (third panel) and anti-c-Myc MAb (fourth panel). (B) HepG2 cells were transfected with pEF1A-HBx-Myc-His_6_. At 48 h after transfection, cells were harvested. Cell lysates were immunoprecipitated with anti-c-Myc MAb or normal mouse IgG control, followed by immunoblotting with anti-Bach1 MAb (top panel) or anti-HBx PAb (second panel). Input samples were immunoblotted with anti-Bach1 MAb (third panel), anti-HBx PAb (fourth panel), and anti-GAPDH MAb (fifth panel). GAPDH served as a loading control. (C) HepG2 cells were transfected with pEF1A-Myc-His_6_ or pEF1A-HBx-Myc-His_6_. At 48 h after transfection, cells were fixed and subjected to indirect immunofluorescence staining with an anti-c-Myc MAb followed by Alexa Fluor 488-conjugated goat anti-mouse IgG (green), and an anti-Bach1 rabbit PAb followed by Alexa Fluor 594-conjugated goat anti-rabbit IgG (red). Nuclei were counterstained with Hoechst 33342 (blue). Scale bar, 10 μm. (D) HepG2 cells were co-transfected with pCAG-FLAG-Bach1 and pEF1A-HBx-Myc-His_6_. At 48 h after transfection, cells were fixed and subjected to an in situ PLA using both anti-Bach1 rabbit PAb and anti-c-Myc mouse MAb. Nuclei were counterstained with Hoechst 33342 (blue). Scale bar, 10 μm.

**Fig. 5:**
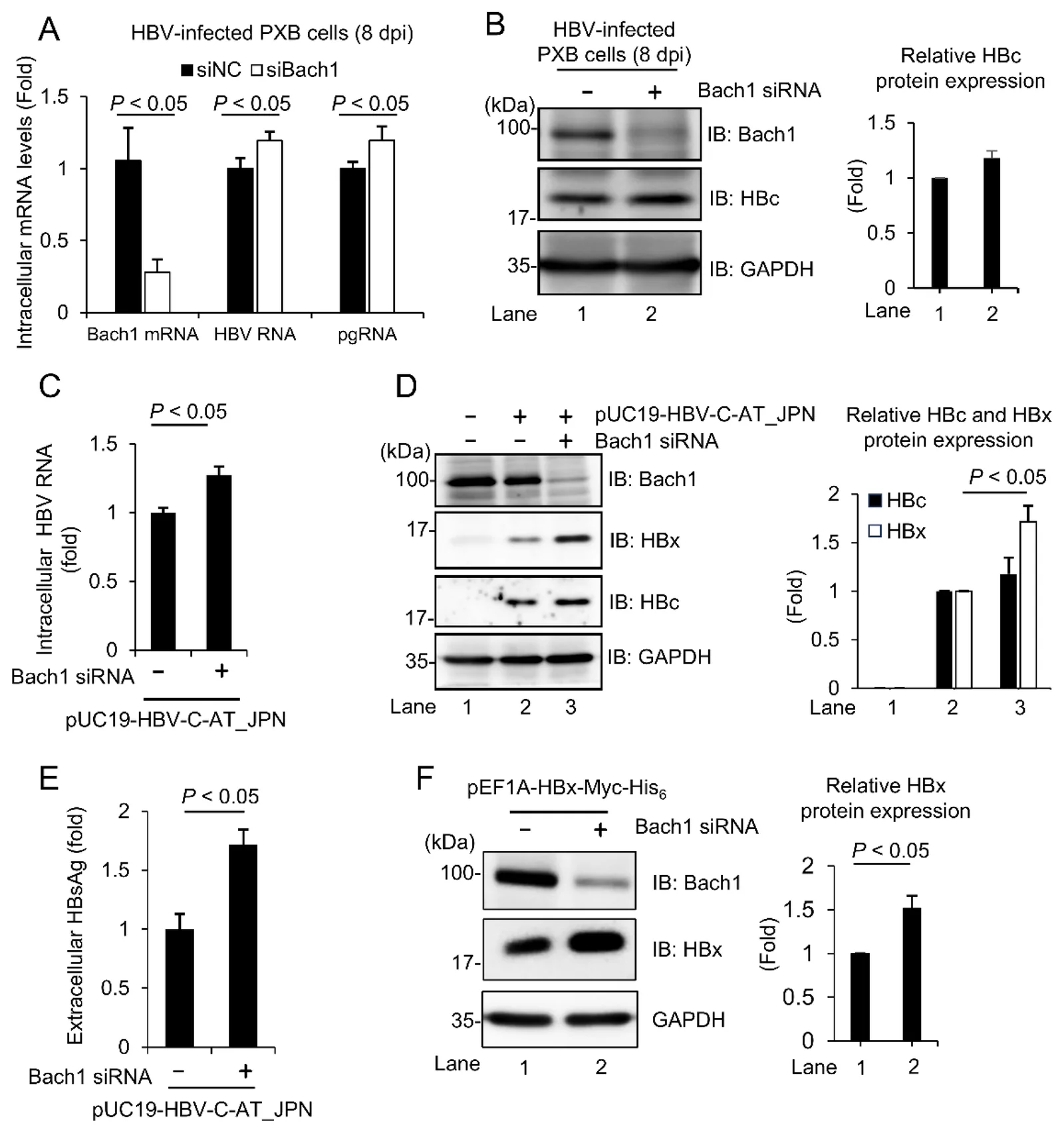
Knockdown of Bach1 enhances HBV replication and increases HBx protein levels (A and B) PXB cells were transfected with 40 nM Bach1 siRNA or control siRNA. At 48 h post-transfection, cells were infected with HBV at 1,000 GEq/cell and harvested at 8 dpi. Total RNA was extracted, and Bach1 mRNA, HBV RNA, and pgRNA were quantified by RT-qPCR (A). Data represent the means ± SEM from three biological replicates. Values for control cells were arbitrarily set to 1.0. (B) Cell lysates were subjected to immunoblotting using anti-Bach1 MAb (first panel), anti-HBc MAb (second panel), and anti-GAPDH MAb (bottom panel), with GAPDH serving as the loading control. The relative levels of HBc were quantified by densitometric analysis from three biological replicates. (C, D, and E) HepG2 cells were transfected with 40 nM Bach1 siRNA or control siRNA. At 24 h after siRNA transfection, cells were transfected with pUC19-HBV-C-AT_JPN. At 48 h after plasmid transfection, the cells were harvested. (C) Intracellular HBV RNA levels were quantified by RT-qPCR. Data represent the means ± SEM from three biological replicates. Values for control cells were arbitrarily set to 1.0. (D) Cell lysates were subjected to immunoblotting using anti-Bach1 MAb (first panel), anti-HBx PAb (second panel), anti-HBc MAb (third panel), and anti-GAPDH MAb (fourth panel), with GAPDH serving as the loading control. The relative levels of HBx and HBc were quantified by densitometric analysis from three biological replicates. (E) Extracellular HBsAg levels were determined by ELISA. Data represent the means ± SEM from three biological replicates. Values for control cells were arbitrarily set to 1.0. (F) HepG2 cells were transfected with 40 nM Bach1 siRNA or control siRNA. At 24 h after siRNA transfection, cells were transfected with pEF1A-HBx-Myc-His_6_. At 48 h after plasmid transfection, the cells were harvested. Cell lysates were subjected to immunoblotting using anti-Bach1 MAb (first panel), anti-HBx PAb (second panel), and anti-GAPDH MAb (third panel), with GAPDH serving as the loading control. The relative levels of HBx were quantified by densitometric analysis from three independent experiments. A P-value < 0.05 was considered statistically significant.

**Fig. 6:**
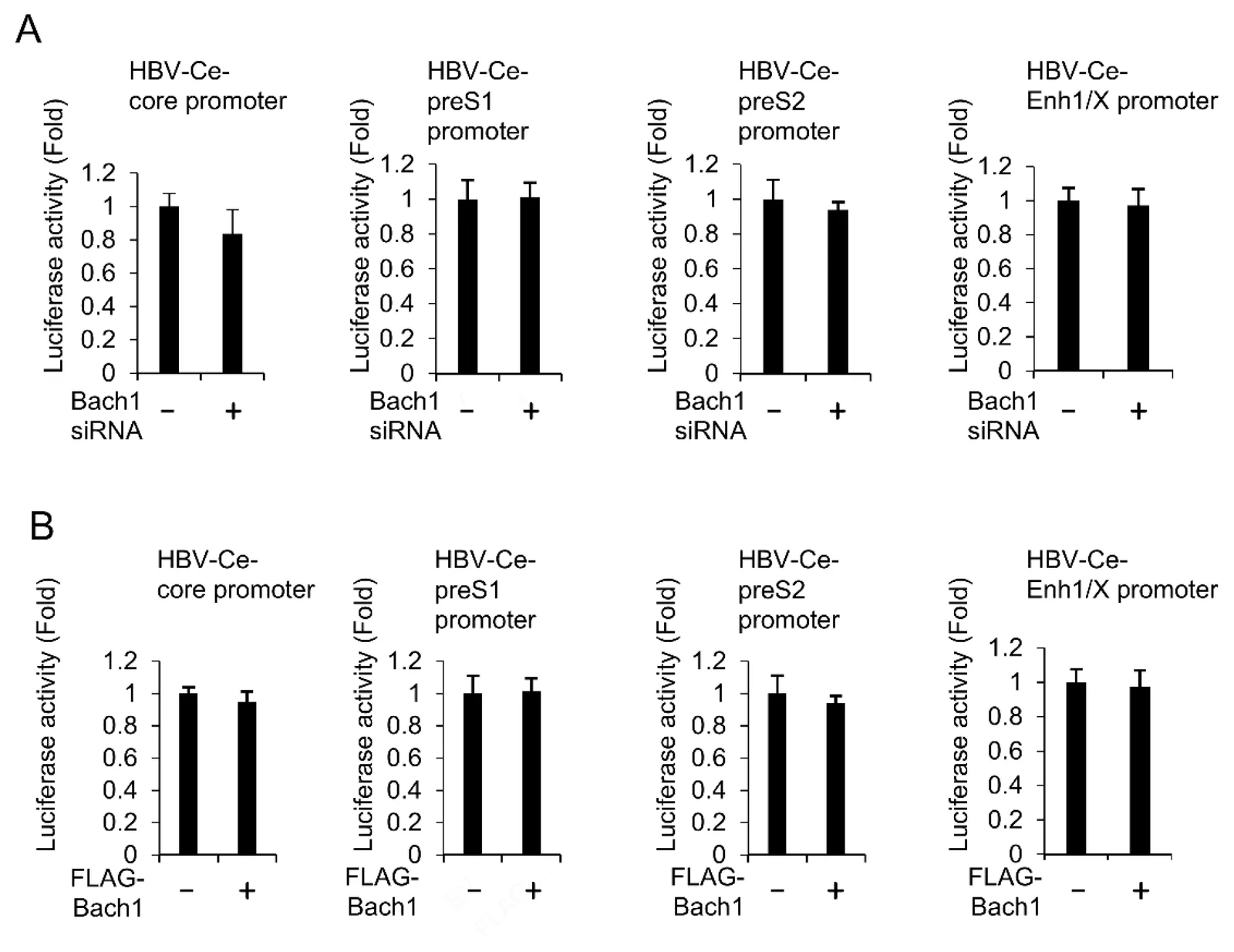
Bach1 does not directly regulate HBV promoter activities HepG2 cells were transfected with 40 nM Bach1 siRNA (A) or pCAG-FLAG-Bach1 (B), together with firefly luciferase reporter plasmids driven by the HBV genotype Ce core, preS1, preS2/S, or Enh1/X promoter. At 48 h after plasmid transfection, luciferase activity was measured and normalized to *Renilla* activity. Data represent the means ± SEM of data from three independent experiments. Value for the control cells was arbitrarily set to 1.0. A P-value < 0.05 was considered statistically significant.

**Fig. 7:**
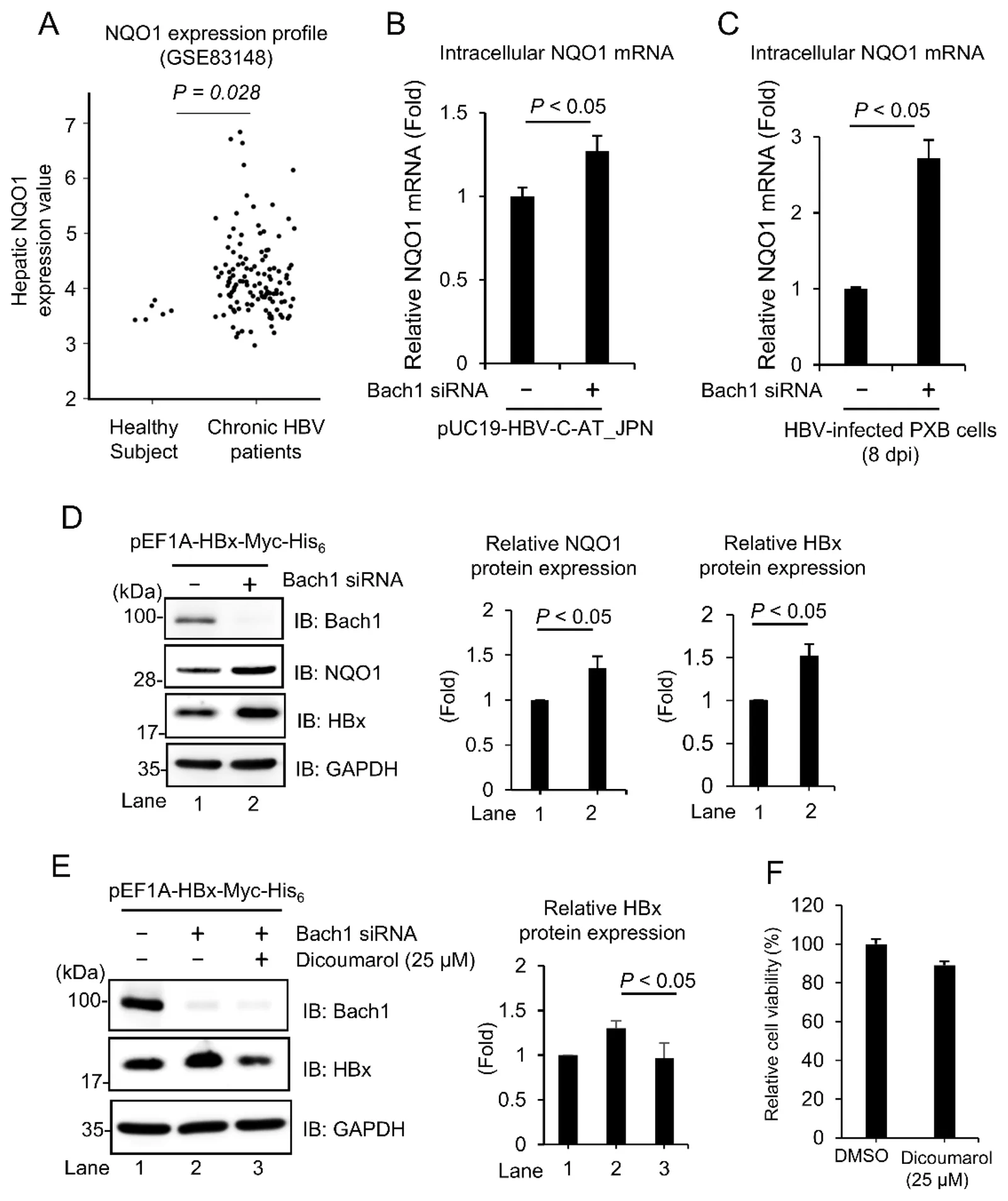
HBV-induced Bach1 downregulation is associated with NQO1 upregulation and HBx stabilization (A) Transcriptomic analysis of liver tissue samples from patients with chronic HBV infection (n = 122) and healthy individuals (n = 6) using the GEO dataset GSE83148. (B) HepG2 cells were transfected with 40 nM Bach1 siRNA or control siRNA. At 24 h after siRNA transfection, cells were transfected with pUC19-HBV-C-AT_JPN. At 48 h after plasmid transfection, cells were harvested. Total RNA was extracted, and NQO1 mRNA was quantified by RT-qPCR. Data represent the means ± SEM from three biological replicates. Values for control cells were arbitrarily set to 1.0. (C) PXB cells were transfected with 40 nM Bach1 siRNA or control siRNA. At 48 h after transfection, cells were infected with HBV at 1,000 GEq/cell and harvested at 8 dpi. Total RNA was extracted, and NQO1 mRNA was quantified by RT-qPCR. Data represent the means ± SEM from three biological replicates. Values for control cells were arbitrarily set to 1.0. (D) HepG2 cells were transfected with 40 nM Bach1 siRNA or control siRNA. At 24 h after siRNA transfection, cells were transfected with pEF1A-HBx-Myc-His_6_. Cells were harvested at 48 h after plasmid transfection. Cell lysates were subjected to immunoblotting using anti-Bach1 MAb (first panel), anti-NQO1 MAb (second panel), anti-HBx PAb (third panel), and anti-GAPDH MAb (fourth panel), with GAPDH as the loading control. The relative levels of NQO1 and HBx were quantified by densitometric analysis from three biological replicates. (E) HepG2 cells were transfected with 40 nM Bach1 siRNA or control siRNA. At 24 h after siRNA transfection, cells were transfected with pEF1A-HBx-Myc-His_6_. At 24 h after plasmid transfection, cells were treated with DMSO or 25 μM dicoumarol for 48 h before harvesting. Cell lysates were subjected to immunoblotting using anti-Bach1 MAb (first panel), anti-HBx PAb (second panel), and anti-GAPDH MAb (third panel), with GAPDH as the loading control. The relative levels of HBx were quantified by densitometric analysis from three biological replicates. (F) Cell viability of HepG2 cells treated with 25 μM dicoumarol for 48 h was assessed using the CellTiter-Glo v2.0 assay. Data represent the means ± SEM from three biological replicates. A P-value < 0.05 was considered statistically significant.

**Fig. 8:**
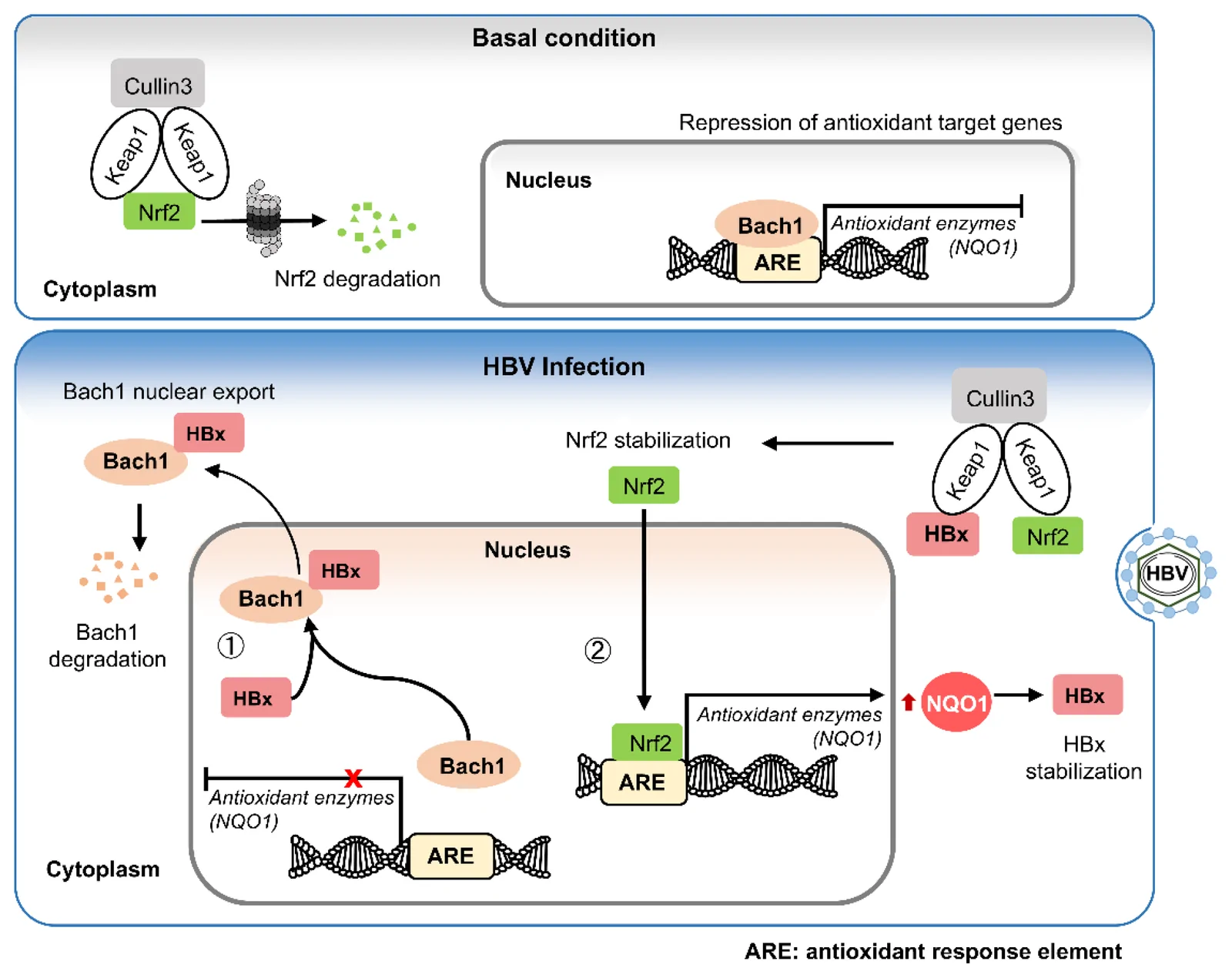
Proposed model of HBx-mediated Bach1 nuclear export and downregulation, leading to Nrf2/ARE activation Under basal conditions, the Cullin3/Keap1 complex targets Nrf2 for ubiquitin-dependent proteasomal degradation, whereas Bach1 localizes to the nucleus and represses the transcription of ARE-driven antioxidant genes. During HBV infection, HBx interacts with Bach1 and promotes Bach1 nuclear export and downregulation (①). In parallel, Keap1 interacts with HBx, leading to Nrf2 activation and nuclear translocation. This coordinated shift in redox regulation favors Nrf2-dependent antioxidant responses (②), resulting in enhanced NQO1 expression and increased stabilization of HBx protein, thereby contributing to HBV replication.

## Data Availability

The dataset analyzed in this study are available from the corresponding author upon reasonable request. Publicly available transcriptomic data were obtained from GEO (GSE83148).
